# Identification of a novel heterozygous SPTB mutation by whole genome sequencing in a Chinese patient with hereditary spherocytosis and atrial septal defect: a case report

**DOI:** 10.1186/s12887-021-02771-4

**Published:** 2021-06-28

**Authors:** Zhanhui Du, Gang Luo, Kuiliang Wang, Zhen Bing, Silin Pan

**Affiliations:** Heart center, Qingdao Women and Children’s Hospital, Qingdao University, 217 Liaoyangxi Road, Qingdao, 266034 China

**Keywords:** Hereditary spherocytosis, Atrial septal defect, SPTB mutation, Whole genome sequencing, Case report

## Abstract

**Background:**

Hereditary spherocytosis (HS) is a common inherited red blood cell membrane disorder characterized by an abnormal increase of spherocytes in peripheral blood. *SPTB* gene mutation is one of the most common causes of HS; however, few cases of HS resulting from *SPTB* mutation in the Chinese population have been reported so far.

**Case presentation:**

A 3-year-old Chinese girl presented to Qingdao Women and Children’s Hospital, Qingdao University, with atrial septal defect (ASD). Meanwhile, she was clinically diagnosed with HS. Whole genome sequencing (WGS) was performed for the proband and her parents for genetic molecular analysis. A novel *SPTB* mutation (c.1756delG) was detected by WGS and confirmed by Sanger sequencing in the proband. This mutation results in a frameshift with a premature termination codon in exon 12, leading to a nonsense mutation (p.Ala586Profs*7). Her parents had no similar symptoms, and blood routine and serum biochemical tests showed no significant abnormalities. The patient’s mother did not know of any relatives with HS-like symptoms. Percutaneous transcatheter closure was successfully performed for treating the ASD.

**Conclusion:**

In this study, we identified a novel *SPTB* frameshift mutation in a Chinese girl with HS. This finding would expand the spectrum of *SPTB* mutations, provide a valuable insight into the genotyping of HS in the Chinese population, and contribute to the clinical management and genetic counseling in HS.

## Background

Hereditary spherocytosis (HS) is a common inherited red blood cell (RBC) membrane disorder characterized by an abnormal increase of spherocytes in peripheral blood. HS occurs in all racial and ethnic groups, and its prevalence in China is about 1.27–1.49 per 100,000 individuals [1]. The HS clinical spectrum ranges broadly from a nearly asymptomatic disease to life-threatening anemia, serious splenomegaly, and/or severe hyperbilirubinemia, even in the same family. About 70% of HS cases are associated with autosomal dominant inheritance, although non-dominant and autosomal recessive modes of inheritance have been described.

HS is mostly characterized by sphere-shaped erythrocytes on peripheral blood smear resulting from qualitative and/or quantitative abnormalities of RBC membrane proteins, which are basically caused by mutations in the corresponding genes. Current evidence indicates that mutations in the ankyrin 1 (*ANK1*; about 50%), spectrin-β, erythrocytic (*SPTB;* approximately 20%), solute carrier family 4, member 1 (*SLC4A*1*;* about 15%), erythrocyte membrane protein band 4.2 (*EPB42*; 10%) and spectrin-ɑ, erythrocytic 1 (*SPTA1*; 5%) are associated with membrane defects in HS [2].

Although *SPTB* mutations mostly cause HS, only few HS families with such mutations have been reported in the Chinese population [3]. Here, we identified a novel *SPTB* frameshift mutation responsible for HS in a Chinese family by whole genome sequencing (WGS).

## Case presentation

### Clinical phenotype

A 3-year-old Chinese girl was referred to Qingdao Women and Children’s Hospital, Qingdao University on April 20, 2017, after a diagnosis of atrial septal defect (ASD) and HS. ASD in this patient was incidentally detected during a physical examination 2 years ago in a local hospital. Subsequent echocardiographic data confirmed this diagnosis, and the girl was suggested to undergo regular check-up. The patient had experienced bouts of weakness and easy fatigability since birth. One month ago, she visited a local hospital for anemia. Based on laboratory findings and the osmotic fragility test, a diagnosis of HS was made. Our hospital was recommended for further treatment.

**Fig. 1:**
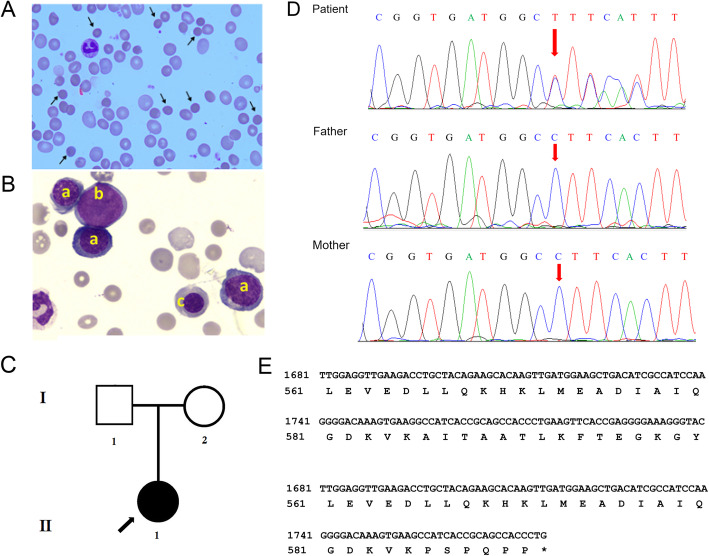
Identification of a novel SPTB frameshift mutation. **A**. Peripheral blood smear of the proband showing moderate spherocytosis (Wright’s-Giemsa staining) (abnormal cells indicated by arrows). **B**. Wright’s-Giemsa staining of the proband’s bone marrow smear demonstrating erythroblastic hyperplasia dominated with rubricytes and metarubricytes: a, intermediate erythroblasts; b, myeloblasts; c, late erythroblasts. **C**. Pedigree of the family with a hereditary spherocytosis (HS) case. **D**. Sanger sequencing confirming the heterozygous mutation of *SPTB* (c.1756delG). **E.** The deletion mutation leads to the formation of a truncated SPTB protein (p.Ala586Profs*7)

On admission, the patient’s sclerae and skin were icteric, and mucosal membranes were pale. Physical examination revealed a systolic ejection murmur with splitting of the second heart sound at the left second intercostal space, and an enlarged spleen in the left subcostal region. Hematologic assessment revealed hemoglobin levels at 8.2 g/dl (normal range, 11–17 g/dl), hematocrit at 23.8% (normal range, 36–56%), RBC count at 3.15 × 10^12^/L (normal range, 3.50–5.30 × 10^12^/L), and white blood cell count at 6.52 × 10^9^/L (normal range, 4.0–10.0 × 10^9^/L). Total serum bilirubin (89.4 μM; normal range, 2.0–22.0 μM), direct bilirubin (11.8 μM; normal range, 0.0–8.0 μM), and indirect bilirubin (77.6 μM; normal range, 0.0–14.0 μM) levels were significantly increased. Liver enzyme levels were normal. RBC size showed disparity, and the presence of spherocytes (about 12%) was noted on peripheral blood smear (Fig. 1A). Bone marrow smear analysis showed active proliferation with erythroid preponderance (myeloid cells was 39.5%, and erythroid cell was 48.5%), increase of intermediate and late erythroblast, different size of mature erythrocyte, and the presence of polychromatic and spherical erythrocytes (Fig. 1B). RBCs showed elevated osmotic fragility. Echocardiography revealed a fossa ovalis ASD with left-to-right shunt, enlarged right atrium and right ventricle, and dilated main pulmonary artery.

The girl was the only child born to healthy non-consanguineous parents (Fig. 1C). Because of “too little amniotic fluid” she was born at 37 weeks of gestation by cesarean section, with a birth weight of 3000 g. It is unclear whether the mother had any specific medication or exposure history during pregnancy. Her parents had no similar symptoms, and their blood routine and serum biochemical tests showed no significant abnormalities. The patient’s mother did not know of any relatives with HS-like symptoms.

The conventional treatment option for ASD is open heart surgery with cardiopulmonary bypass, which would put the HS patient at risk of hemolytic anemia because of the deleterious effects of the heart-lung machine. Hence, percutaneous transcatheter closure for ASD was successfully performed. Because the patient lived in another province, there was no follow-up after discharge on May 7, 2017.

### Molecular analysis

Genetic molecular analysis was performed for the proband and her parents to assess the genetic cause of HS. The study protocol was approved by the Ethics Committee of Qingdao Women and Children’s Hospital, Qingdao University. Written informed consent for clinical and molecular analyses was obtained from all subjects.

WGS was performed by BGI (Shenzhen, China) following the standard procedure. Sequencing data were filtered by removing low-quality reads, adapters, and sequences with more than 5% unknown bases. Clean reads were matched to human reference genome 19 (hg19) using the Burrows-Wheeler Alignment tool. Duplications were marked with Picard (http://broadinstitute.github.io/picard) and BAM was sorted with Samtools2. GATK Best Practices Pipeline, which includes Local realignment around indels, Base quality score recalibration (BQSR), Haplotype Caller, and Variant Quality Score Recalibration (VQSR), was used in the process of SNP and INDEL calling. After filtering high-quality variants, SNPs and INDELs were annotated using the snpEff 3, dbSNP (version 147), 1000 genomes (August 2015), ExAC (version 3), and dbNSFP (version 2.9) databases.

A total of 20,154 sequence variants were identified and subjected to interpretation, according to the variant interpretation guidelines of the American College of Medical Genetics and Genomics (ACMG) [4]. All the identified variants were firstly filtered using the dbSNP, HapMap, 1000 project, and 100 Chinese healthy adults’ local databases, deleting all variants with frequency > 1% in the healthy population. Then, all the remaining filtered variants were searched in the OMIM and CGD databases for identifying the variants of genes associated with the disease phenotype. Then, the selected variants were further searched in published literatures in Google scholar, PubMed and the Human Gene Mutation Database (HGMD) [5]. The identified variants were also predicted by in silico software such as SIFT, Polyphen-2, Mutation Taster, and PROVEAN. The classification of the newly identified variants was based on the ACMG Guidelines [4].

Sanger sequencing was performed for validating the variants identified by WGS. Primers were designed based on reference human genome sequences in NCBI-GenBank. Amplification was performed on an ABI 9700 Thermal Cycler (Applied Biosystems, Foster City, CA, USA). The amplified products were subjected to direct sequencing on an ABI PRISM 3730 automated sequencer (Applied Biosystems). DNASTAR SeqMan (DNASTAR, Madison, Wisconsin, USA) was used for comparing and analyzing the sequencing data. The primer sequences were: F, 5′-TCCAAGTTGGGTTGTTAGGC-3′; R, 5′-GTTCTCTTTGCCAGGCTCAC-3′.

As a result, a heterozygous variant c.1756delG was identified in exon 12 of the *SPTB* gene in the proband and confirmed as a de novo variant because Sanger sequencing of her parents showed a wild type (Fig. 1D). The pedigree of the proband was shown in Fig. 1C. This mutation causes a frameshift by the formation of a premature stop codon. This frameshift mutation leads to the synthesis of a truncated SPTB protein (p.Ala586Profs*7) comprising 593 amino acids instead of 2137 amino acids in the wild type counterpart (Fig. 1E). The *SPTB* variant is predicted as deleterious, probably damaging, disease causing and deleterious by SIFT, Polyphen-2, MutationTaster, and PROVEAN and a high frequency of de novo mutations being reported in the *SPTB* gene (Table 1). Hence, it is a *loss-of-function* mutation. Sanger sequencing revealed that this mutation was novel, which was not present in the proband’s unaffected parents, 100 other healthy individuals, ExAC, HGMD or 1000 Genome databases. Hence, a novel de novo *SPTB* mutation c.1756delG was confirmed in the Chinese girl. All data used for the analysis in this study are available in the CNGB Nucleotide Sequence Archive with accession number CNP000089 (CNSA: https://db.cngb.org/cnsa).

**Table 1:** Previously reported hereditary spherocytosis cases with SPTB mutations

Author & year	Number of Patients	Exon	Nucleotide	Amino acid	Type	Mutation type
Park 2016 [6]	1	5	c.624_640delinsACCTCC	p.Phe208Leufs*12	Heterogeneous	Frameshift
	2	11	c.1493_1502delGCAT CACGGC	p.Arg498Profs*72	Heterogeneous	Frameshift
	1	12	c.1795 + 2_1795 + 3delTG		Heterogeneous	Splicing error
	1	13	c.2572_2573delGA	p.Asp858Argfs*3	Heterogeneous	Frameshift
	1	14	c.2686G > T	p.Glu896*	Heterogeneous	Nonsense
	1	15	c.3058C > A	p.Gln1020Lys	Heterogeneous	Missense
	1	15	c.3205delG	p.Asp1069Ilefs*59	Heterogeneous	Frameshift
	1	15	c.3440delA	p.Asp1147Valfs*79	Heterogeneous	Frameshift
	1	18	c.3976G > T	p.Glu1326*	Heterogeneous	Nonsense
	1	20	c.4291C > T	p.Arg1431*	Heterogeneous	Nonsense
	1	25	c.5266C > T	p.Arg1756*	Heterogeneous	Nonsense
Xue 2020 [7]	1	19	c.4181G > A	p.W1394X	Heterogeneous	Nonsense mutation
	1	2	c.211G > A	p.V71M	Heterogeneous	Missense mutation
	2	23	c.4973 + 5G > A	Splicing	Heterogeneous	Splice mutation
Meglic 2020 [8]	7	Unspecified	c.4796G > A	p.Trp1599Ter	Heterogeneous	Nonsense mutation
Xue 2019 [9]	2	23	c.4973 + 5G > A	splicing	Heterogeneous	Splice mutation
	1	19	c.4181G > A	p.W1394X	Heterogeneous	Nonsense mutation
	1	2	c.211G > A	p.V71M	Heterogeneous	Missense mutation
van Vuren 2019 [10]	17	Unspecified	Unspecified	Unspecified	Unspecified	Unspecified
Li 2019 [11]	1	Unspecified	c.2413 C > T	p.Gln805*	Heterogeneous	Nonsense mutation
Shen 2019 [12]	1	23	c.4873 C > T	p.R1625X	Heterogeneous	Nonsense mutation
Choi 2019 [13]	28	Unspecified	Unspecified	Unspecified	Unspecified	Unspecified
Shin 2018 [14]	1	13	c.1956G > A	p.Trp652*	Heterogeneous	Nonsense mutation

## Discussion and conclusions

This study described a Chinese family with a member affected by HS. A novel *SPTB* mutation (c.1756delG) was detected in the proband by WGS, and confirmed by Sanger sequencing. This mutation results in a frameshift with a premature termination codon within exon 12, leading to a nonsense mutation (p.Ala586Profs*7).

The *SPTB* gene is located on chromosome 14q23.3, and encodes the β-spectrin protein, which is typically composed of 4 structural domains, including the activity binding domain, dimerization domain, spectrin repeats, and ankyrin binding domain. β-spectrin forms the cytoskeleton along with ɑ-spectrin, and tethers it to the RBC membrane by interacting with ankyrin to maintain erythrocyte deformability and stability. *SPTB* mutation is the second most common pathologic mutation in HS, only after *ANK1* mutation [2]. A recent trial reported 6 frameshift, 5 nonsense and 1 splicing error mutations in the *SPTB* gene in patients with HS [6]. Another study assessing 35 Chinese patients with suspected HS reported 3 cases with mutated SLC4A1, 16 with ANK1 mutations, and 16 with mutated *SPTB*, indicating that *SPTB* and *ANK1* are the most commonly mutated genes in Chinese HS patients [15]. In our study, DNA analysis of the patient revealed a G deletion at the position 1756 that resulted in a frameshift mRNA by premature termination codon within exon 12. This premature termination codon-bearing transcript might be degraded by nonsense-mediated mRNA decay [7] or produce a C-terminal truncated protein, either of which might lead to a β-spectrin deficiency. Deficient SPTB protein levels due to frameshift mutation in the spectrin repeats domain are considered a cause of HS. The current patient showed increased osmotic fragility, which is a well-known pathological feature of type 2 HS caused by *SPTB* mutation. Previously reported hereditary spherocytosis cases with *SPTB* mutations were summarized in Table 1 [8–14, 16].

Similar to patients with other hemolytic anemia types, HS cases are subject to various problems such as severe splenomegaly with or without gallbladder disease. In this report, the proband showed hepatosplenomegaly. Splenectomy is a very effective treatment for reducing hemolysis, leading to significantly prolonged red cell lifespan. However, it should be performed only after careful risk-benefit assessment. The current patient was 3 years old, and it is not recommended to perform splenectomy before cardiac surgery since the immune function should be maintained. Additionally, the patient was diagnosed with ASD. Patients with concurrent ASD and HS are rare. To the best of our knowledge, only four such cases have been reported [17–20]. Percutaneous transcatheter closure for ASD was performed to avoid potential risk of hemolytic anemia.

HS diagnosis is made on clinical suspicion often including a family history and confirmatory blood test. However, about 25% of patients have a negative family history, which will increase the difficulty of HS diagnosis [21]. Confirming hereditary RBC membrane disorders at the molecular level using next-generation sequencing is important for HS diagnosis, clinical management as well as genetic counseling. WGS enables the screening of all coding regions and deep intronic variants usually causing disease. In this study, a novel *SPTB* mutation responsible for HS was successfully detected using WGS in a Chinese patient. These results expand the spectrum of *SPTB* mutations, providing novel insights into the molecular mechanisms involved in HS and confirming WGS as an effective method for identifying novel pathogenic mutations.

## Data Availability

The datasets used and/or analyzed during the current study are available from the corresponding author on reasonable request.

## Abbreviations

HS: Hereditary spherocytosis
ASD: Atrial septal defect
WGS: Whole genome sequencing
RBC: Red blood cell
ANK1: Ankyrin 1
SPTB: Spectrin-β
BQSR: Base quality score recalibration
VQSR: Variant Quality Score Recalibration
ACMG: American College of Medical Genetics and Genomics
HGMD: Human Gene Mutation Database
